# Microstructure and Residual Stresses of AA2519 Friction Stir Welded Joints under Different Heat Treatment Conditions

**DOI:** 10.3390/ma13040834

**Published:** 2020-02-12

**Authors:** Lucjan Śnieżek, Robert Kosturek, Marcin Wachowski, Bogusz Kania

**Affiliations:** 1 Faculty of Mechanical Engineering, Military University of Technology, 2 gen. S. Kaliskiego St., 00-908 Warsaw, Polandmarcin.wachowski@wat.edu.pl (M.W.); 2 Institute of Metallurgy and Materials Science of Polish Academy of Sciences, 25 Reymonta St., 30-059 Kraków, Poland; bogusz.kania@gmail.com

**Keywords:** friction stir welding, aluminum alloy, AA2519, heat treatment, microstructure, residual stresses

## Abstract

The aim of this research was to investigate the effect of different heat treatment conditions of AA2519 friction stir welded joints on their microstructure and residual stresses. The following welding parameters have been used: 500 rpm tool rotation speed, 150 mm/min tool traverse speed, tool tilt angle 2°, pressure force 17 kN. The welded material was investigated in three different configurations: HT0, HT1, and HT2. The first type of weld (HT-0) was made using AA2519 alloy in non-precipitation hardened state and examined in such condition. The second type of weld (HT-1) has been performed on AA2519-T62, that corresponds to precipitation hardening condition. The last type of weld (HT2) was performed on annealed AA2519 and the obtained welds were subjected to the post-weld precipitation hardening process. The heat treatment was carried out in two stages: solution heat treatment (530 °C/2 h + cooling in cold water) and aging (165 °C/1 0 h). Residual stresses were measured using X-Ray diffraction patterns obtained from Bruker D8 Discover X-ray diffractometer utilizing the concepts of Euler cradle and polycapillary primary beam optics. The conducted research indicates that the best material properties: homogenous microstructure and uniform distribution of microhardness and compressive state of residual stresses were obtained for the HT-2 series samples subjected to heat treatment after the friction stir welding (FSW) process.

## 1. Introduction

The reduction of energy consumption, especially in the automotive and aerospace industry is one of the greatest challenges facing modern civilization. This task can be accomplished by replacing the previously used materials with their lighter counterparts and introducing new technologies in the production process [1,2]. A visible example of this trend is the wide use of aluminum alloys, especially in the aerospace and automotive industries [3,4,5,6,7]. However, technological problems with welding limit the field of applications of these materials. A large part of aluminum alloys, including high-strength aluminum alloys of the 2xxx and 7xxx series, are considered difficult to weld by conventional methods. The friction stir welding (FSW) method is meeting these difficulties. In this technique, the rotating tool is inserted into the contact zone of the pressed elements to such a depth that there is friction between the shoulder and the surface of the joining elements [8]. As a result of temperature increase at the spot of interaction, the material under consideration is plasticized. The tool moving along the contact line successively mixes the plasticized material from the neighboring elements to form a joint. With optimized process parameters, a weld is obtained, within which the mechanical mixing of the joined materials occurs without reaching their melting point. Rotating and traversing tool results in the formation of equiaxial, dynamically recrystallized, ultrafine grains in the stir zone. The FSW process is used increasingly due to several advantages. The most important ones include improved mechanical properties of the obtained joints, especially in terms of fatigue strength, dimensional stability of workpiece resulting from a significant reduction of residual stresses, and its more favorable distribution when compared to the conventional fusion welding methods [9]. The FSW method has been successfully used to join not only aluminum alloys but also to join steel and titanium alloys [10,11,12]. Previous studies of aluminum joints produced by FSW have shown that the microstructure and mechanical properties strongly depend on the heat treatment conditions before and after the process. The influence of heat treatment is also reflected in the values of material stresses in the welded joint [13,14,15,16,17]. The material investigated in this paper is a modification of AA2519 by the addition of 0.36% scandium. It is a high-strength aluminum alloy, used primarily in military applications. Due to its excellent properties, such as high specific strength, high ballistic resistance, and fracture toughness, it is used for the production of military helicopters, fighter aircraft, and advanced amphibian assault vehicles. The Sc-modified version of this alloy is currently under research by authors for its fatigue properties and joining by FSW [18,19,20]. Due to high copper concentration, AA2519 is quite difficult to fusion weld including a high risk of hot cracking. Additionally, AA2519 is a precipitation-hardening alloy, so it is very sensitive to an elevated temperature due to thermally unstable θ′ strengthening phase [21]. While FSW is a process characterized by a lower heat input during the formation of a weld in comparison to traditional welding, it allows limited strengthening phase dissolution, and overaging. Although the amount of heat generated during the FSW process is relatively small, the thermal cycle still can lead to coagulation, dissolution and overaging of the strengthening phase [19]. In the case of precipitation-hardened alloys, strength and hardness depend on the size and distribution of the precipitates. Essentially, the loss of strength after the FSW process can be recovered by a suitable heat treatment. Several studies have been devoted to the impact of joining and heat treatment parameters on the quality characteristics of the joint [22,23,24,25]. However, only a few are published referring directly to the AA2519 alloy [26,27,28]. Thus, the presented work focuses on the results of FSWed AA2519 in terms of microstructure analysis and distribution of residual stresses depending on the condition of the material, that is, the applied heat treatment (precipitation hardening) before and after the joining process. Mishra and Ma discussed the issue of the distribution of residual stresses in FSW joints of aluminum alloys [29]. Based on the publications, it was found that the residual stresses generated in the FSW process are significantly lower compared to the stresses generated using conventional welding methods [30,31,32,33]. Donne et al. reported that the maximum values of longitudinal stresses are greater than transverse stresses and do not exceed 100 MPa [31]. Peel et al. discovered that the distributions of these stresses take the form of the letter “M”, in which the maximum tensile stresses are located at a distance corresponding to the radius of the tool shoulder from the axis of the joint, i.e. in the heat affected zone (HAZ) [33]. On the basis of comparative studies, it was found that the values of residual stresses obtained by the methods of cut compliance, X-ray diffraction, neutron diffraction, and high energy synchrotron radiation are comparable. De Giorgi et al. emphasized the significant influence of the tool shape on both the residual stresses distributions and the fatigue properties of AA6082-T6 alloy joints obtained with flat, concave and spiral pins [34]. The fatigue strength of the samples made with the tool with the concave and flat surface were almost identical and lowered by about 15%–20% than samples from the base material. In the case of samples made with a tool with a spiral resistance surface, fatigue strength was significantly lower. Fratini and Zuccarello investigated the FSW joints of four different aluminum alloys with three different sets of parameters of the joining process used for each alloy: with a low, average and high specific thermal contribution [35]. Different stress distributions were obtained into the joint depending on the aluminum alloy grade, however no correlation was found between the changes of these stresses and the level of the applied specific thermal contribution. The impact of both the residual stresses and the parameters of the bonding process on the mechanical properties of the tested alloys was investigated. Bussu and Irving presented a strong relationship between the crack propagation rate and its direction in relation to the FSW joint line in the 2024-T351 alloy [36]. This relationship was correlated with the distribution of residual stresses in the vicinity of the joint. It was observed that 2% plastic deformation in the direction perpendicular to the joint line reduces the residual stresses in the joint. The crack propagation rate in the samples prepared in this way was almost identical to that in the base material regardless of the location and direction of the fracture. The distribution of residual stresses was determined by the cut compliance method described in [37,38]. Fratini et al. investigated the influence of residual stress on fatigue crack growth in 2024-T351 friction stir welded joints [39]. A typical distribution of longitudinal tensile stresses was obtained. The maximum values of these stresses ranging from 50 to 60 MPa corresponded to the limit defined by the surface of the tool resistance, i.e. the minimum microhardness zone. In the stir zone, tensile stresses were estimated at about 40 MPa. A relationship was found between the rate of fatigue cracking and the distribution of stresses and microhardness. The lowest cracking velocity was observed in the zone with maximum compressive stresses. In turn, Lemmen et al. studied the impact of the location of the notch in the form of a 1 mm diameter hole on the so-called fatigue initiation for the aluminum alloy 2024-T3 [40]. The results for samples loaded in the direction of the weld axis (LT) depends strongly on the location of the notch in relation to the axis of the FSW weld. For notches at a distance of 10 and 12 mm from the weld axis, the results were comparable and better in relation to the results for the base material. At the distance of 7 mm from the weld axis, the higher values of mean stress at notch were obtained. The lowest results were recorded for samples with the notch distant by 7.5 mm from the axis of the weld, i.e. in the place of the maximum tensile stresses. No significant effect of microstructure on fatigue life of notched samples was noticed.

## 2. Materials and Methods

The studies were carried out on samples made of AA2519. The material was delivered in the form of sheets with 5 mm thickness. The chemical composition of the material was established by producer (Institute of Non-Ferrous Metals, Light Metals Division in Skawina, Poland) and is shown in Table 1.

FSW joints were made using sheets of dimensions 5 × 100 × 400 mm. The following welding parameters have been used: 500 rpm tool rotation speed, 150 mm/min tool traverse speed, tool tilt angle 2°, pressure force 17 kN. The parameters have been selected based on authors experience to provide the lowest affection of heat on the welded elements. The joining process was carried out in the pressure control mode, and the joint was made with ESAB Legio 4UT device using MX Triflute tool with dimensions presented in Table 2.

The welded material was investigated in three different configurations: HT0, HT1, and HT2. The first type of weld (HT-0) was made using AA2519 alloy in non-precipitation hardened state and examined in such condition. The second type of weld (HT-1) has been performed on AA2519-T62, what corresponds to precipitation hardening condition. The last type of weld (HT2) was performed on annealed AA2519 and the obtained welds were subjected to the post-weld precipitation hardening process. The heat treatment was carried out in two stages: solution heat treatment (530 °C/2 h + cooling in cold water) and aging (165 °C/10 h). These parameters of the heat treatment allow to obtain the precipitated hardened alloy, strengthen by θ’ phase and designated as AA2519-T62. The designations and states of samples are presented in Table 3.

**Figure 1:**
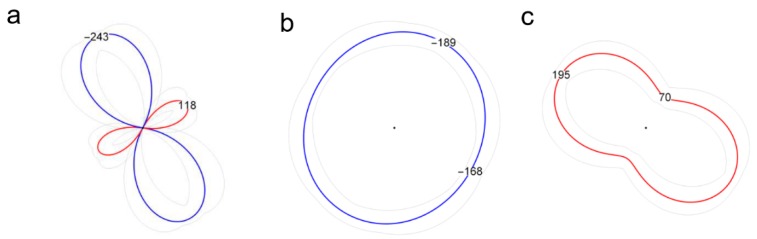
The graph showing the surface stress tensor (σ_3_ ≈ 0) with a compressive squeezing principal component σ_1_ = −243 ± 12 MPa, acting in the direction γ = 63 ± 13° from the horizontal direction (direction of the clockwise angle) and perpendicular to it, the tensile component σ_2_ = 118 ± 15 MPa (**a**). The graph of the compressive surface stresses close to the isotropic situation σ_1_ = σ_2_ (main stresses of equal value) (**b**). The graph of anti-isotropic anisotropic tensile stresses with principal values σ_1_ = 195 ± 14 MPa, σ_2_ = 70 ± 17 MPa and the σ_1_ acting direction γ = 32 ± 10° from the horizontal direction (**c**).

Microhardness measurements using the Vickers method were performed using the Struers Dura Scan 70 device. For each sample, measurements were taken along the cross-section of the joint at a distance of 2.5 mm from the root face. A load of 0.98 N was applied for 10 s, placing the measurement points every 0.5 mm. The microhardness measurements have been performed one month after the joining process, what allows to stabilize the properties of precipitation hardening alloy weld [41]. Metallographic observations were carried out on samples cut from welds in a direction perpendicular to the direction of joining. The samples were cut using the wire electrical discharge machining (WEDM) method. All samples used for the experiments were polished using a 3 and 1 μm diamond suspensions and then etched with the Keller (20 mL H2O + 5 mL HNO3 + 2 mL HF + 1 mL HCl) reagent for 10 s. Observations of the base material and the obtained welds were carried out using the Olympus LEXT OLS4100 confocal laser scanning microscope. Residual stresses were measured using X-ray diffraction patterns obtained from Bruker D8 Discover X-Ray diffractometer utilizing the concepts of Euler cradle and polycapillary primary beam optics. The investigation involved determination of directional stresses σ_ϕ_ based on the d_hkl_ = d_hkl_(sin^2^ψ) relation, measured in four directions on the polished cross-sections of the joints at each measurement point, and estimation of the in-surface stress state [42]. The observed diffraction reflection was the Al peak at the angular position 2θ = 94.3° for the applied Co Kα radiation. In the stress tensor calculations, the isotropic elastic constants E_Al,311_ = 68.5 GPa, ν_Al,311_ = −0.35 were used. The measured data were interpreted and visualized using the TARSIuS (Texture-Aided Residual Stress Investigation System) package, using polar diagrams of surface stress values (Figure 1) [42].

**Figure 2:**
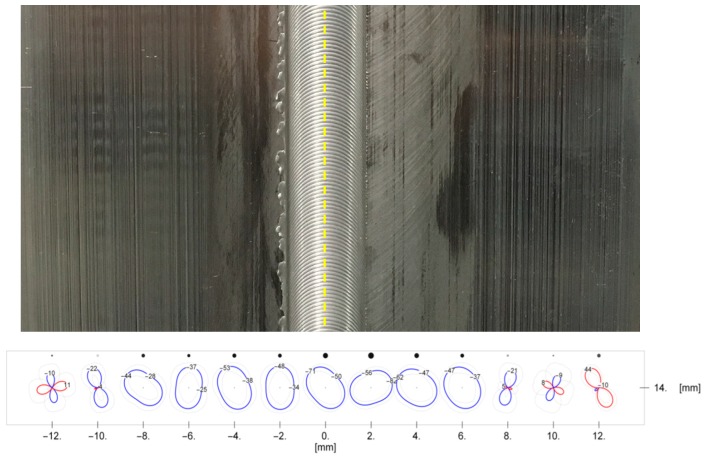
The distribution of the measuring points on the surface of the HT-0 FSW joint. Line of the weld marked with yellow dashed line.

The position of the measuring points in relation to the weld line is presented in Figure 2. Black discs over polar diagrams of surface stresses represent the relative value of the determined principal stresses at a given measurement point. The higher the absolute stress value, the more visible disc.

The main aim of this investigation is to determine optimized heat treatment for more favourable microstructure and residual stress distribution.

## 3. Results and Discussion

**Figure 3:**
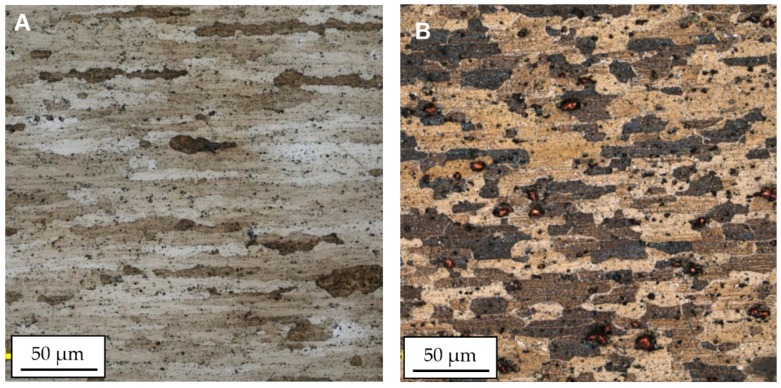
Microstructure of AA2519 alloy in the HT-0 condition (**A**) and in the HT-1 condition (**B**).

The image of the microstructure (material in the HT-0 and HT-1 condition) is presented in Figure 3. The microstructure of AA2519 aluminum alloy is characterized by elongated grains oriented parallel to the rolling direction and it consists of Al matrix and Al_2_Cu precipitates.

**Figure 4:**
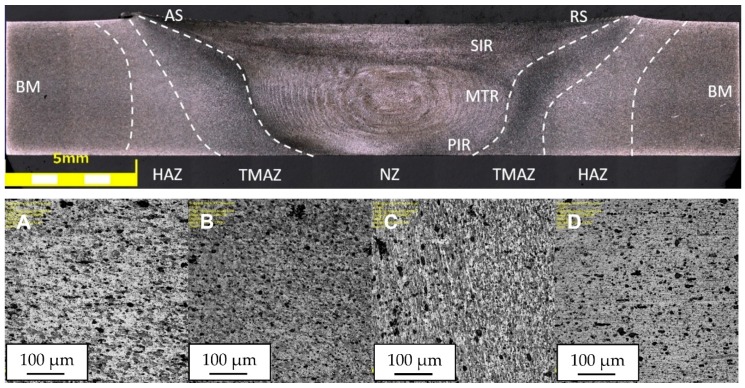
The microstructure of the FSW joint from the AA2519 alloy in the HT-0 condition; (**A**,**B**) the NZ areas marked in the photo, (**C**) the boundary between NZ and TMAZ, (**D**) HAZ.

**Figure 5:**
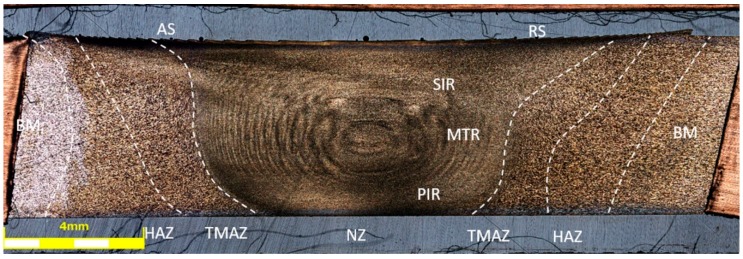

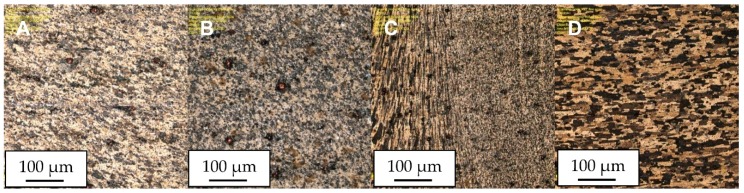
The microstructure of the FSW joint from the AA2519 alloy in the HT-1 condition; (**A**,**B**) the NZ areas marked in the photo, (**C**) the boundary between NZ and TMAZ, (**D**) HAZ.

**Figure 6:**
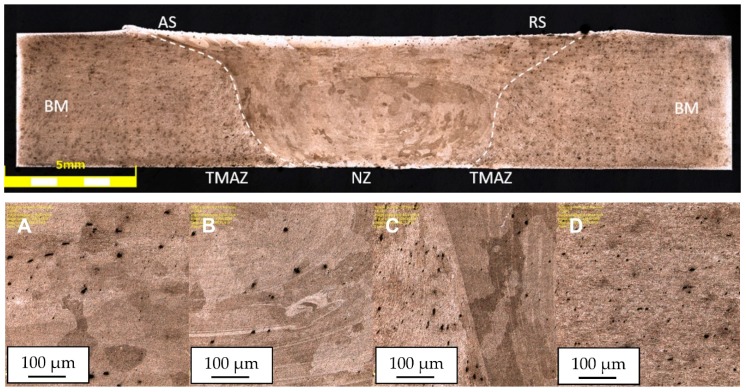
The microstructure of the FSW joint from the AA2519 alloy in the HT-2 condition; (**A**,**B**) the NZ areas marked in the photo, (**C**) the boundary between NZ and TMAZ, (**D**) HAZ.

The obtained joint quality depends on the properties of the material being welded and the parameters of the process, determining the amount of heat generated and the flow of plasticized material. Due to the severe plastic deformation of the material and the temperature impact, three zones, differing in their microstructure, can be distinguished. These are: nugget zone (NZ) with ultrafine, dynamically recrystallized grains; thermo-mechanical affected zone (TMAZ), characterized by deformed grains and partially recrystallized microstructure; and the heat affected zone (HAZ) formed only by the influence of heat in the vicinity of the joint. The joint area additionally delimits the upper shoulder influenced region (SIR), the mid-thickness region (MTR), the lower pin influenced region (PIR). Due to the unsymmetrical flow of material around the tool during the welding process, the advancing side (AS) should be specified, localized on the side of the material, in which the direction of rotation of the tool is same as the translation of the tool, and the retreating side (RS), in which the rotation and translation are in opposite direction. The arrangement of the respective areas is shown in Figure 4, Figure 5 and Figure 6, presenting the cross-sections of the joints in the states: HT-0 (Figure 4), HT-1 (Figure 5), HT-2 (Figure 6), perpendicular to the direction of the movement of the tool. In addition to the differences in the microstructure, these areas have separate values of hardness and residual stresses. The widths and properties of the welding zones can vary significantly depending on the parameters used for the joining process [43,44]. The used welding parameters also affect the presence of defects in the joint. In all tested joints, no defects were found, including tunnel defects, characteristic for FSW joints and possibly extending over the entire length of the weld. The NZ, TMAZ and HAZ zones in the HT-0 (Figure 4) and HT-1 (Figure 5) reveal small, recrystallized grains distributed evenly in all tested joints compared to the base material. It was observed that the grain size differs for each microstructural zone.

**Figure 7:**
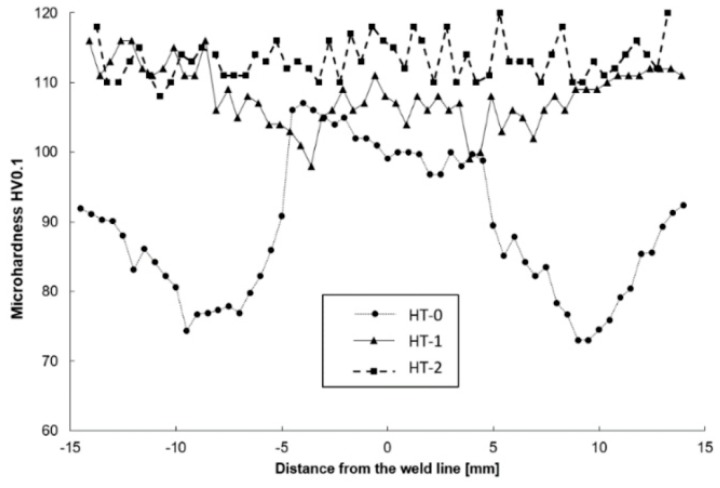
Microhardness of cross-sections of FSW welds.

The weld nugget zone (NZ) in all tested joints is characterized by the structure of the “onion rings”, within which the rings are located at a precisely defined distance from each other, depending on the used parameters of the joining process. The nugget zone localized in the central part of the weld extends in the vertical orientation from the central region (MTR) to the area of weld of the lower part of the pin (PIR). The weld nugget zone is a region formed by direct passage of the tool tip through the material and therefore experiences high temperature, severe plastic deformation and is characterized by a dynamically recrystallized, fine-grained structure in HT-0 and HT-1 joints, with hardness values between 98 and 111 HV0.1 (Figure 7). The final grain size in NZ is strongly dependent on the thermal cycle of FSW process. The NZ zone in the HT-2 state is characterized by grains of a relatively large size, which is the result of grain overgrowth due to the heat treatment applied after the joining process. The plastic deformation, generated by the movement of the tool and the intense heating of the plates within the joint as a result of friction, promotes dynamic recrystallization in the weld nugget zone [25]. The research performed by Durdanovic et al. has shown that almost 80% of the total heat is generated between the tool shoulder and the surfaces of the joined plates resulting in different grain sizes [45]. This is the reason for differences in the size of the grain within the nuggets. The grain size is reduced from the upper surface towards the lower region of the nugget zone (PIR). This relation was observed for the HT-0 and HT-1 welds. As a result of the plastic deformation and the friction generated between the pin and the welded plates, the temperature in the NZ increases to (0.6–0.9) Tm, where Tm is the melting temperature of AA2519 [25]. The heat generated is not evenly distributed in the stirring zone. Due to the high thermal conductivity of aluminum, the heat transfer rate to the substrate plate and environment is relatively large, preventing PIR temperature to rise, causing the grains to be smaller at the bottom of the weld than at the center or top. It is assumed that this is a result of differences in the level of heat generated, as a function of the distance from the tool’s shoulder to the end of the pin. The structural changes resulting from the joining process are reflected in the microhardness distributions. The obtained microhardness distributions (Figure 7) show different tendencies for the tested joints. Only for the HT-2 state, the microhardness distribution shows a linear characteristic, which remains at the level of 108-120 HV0.1. This should be explained by the heat treatment process that the joint was subjected to, resulted in strengthening of the material with θ′ phase and full recrystallization of the stir zone. This investigation is confirmed by the observations of the microstructure of the joint, which do not show significant differences in grain size in particular joint areas. Such differences are clearly visible in the case of the HT-0 and HT-1 samples. The effect of joining on the TMAZ microstructure is smaller than in the NZ zone, however, to a certain extent the influence of the rotational flow of the plasticized material on the formation of elongated grains in the microstructure of this zone is visible. The advancing side (AS) is characterized by a sharp border between NZ and TMAZ for all tested joint states. The retreating side (RS) is characterized by a more complex microstructure, with no clear boundary between NZ and TMAZ (Figure 4, Figure 5 and Figure 6). The degree of grain deformation is higher on the AS, while gradual deformation of grains is observed on the RS. In TMAZ zones of the HT-0 and HT-1 joints, a highly deformed structure with characteristic of elongated grains was observed. The TMAZ of the HT-2 joint is characterized by the presence of equiaxial grains as a result of the heat treatment. The HT-0 joint reveals a broader TMAZ in both the AS and RS compared to the HT-1 joint. The estimated TMAZ width is 2.5 mm for the HT-0 joint and 1.0 mm for the HT-1. The FSW studies of the AA2519 alloy performed by Fonda and Bingert [46] showed the lowest joint hardness for the TMAZ area, as confirmed by our research (Figure 7). Liu showed that TMAZ was the weakest zone in the joints made using the FSW method of aluminum alloy AA2519, and the fracture at tensile test occurred exactly in the TMAZ [47]. For joint in the HT-0 state, the microhardness reaches a maximum (108 HV0.1) at the point of transition between the NZ and TMAZ, which is related to significant grain refinement resulting in the strengthening of the material. The observed decrease in microhardness is caused by the grain growth of the bonded material and is most visible at the border between the TMAZ and HAZ zones. For the HT-1 joint, the measured value of the microhardness of the nugget zone is in the range of 104-111 HV0.1 and is slightly lower than the microhardness value of the base material of approx. 115 HV0.1. The slight decrease in microhardness in this area of the nugget zone should be explained by the dissolution of the alloy strengthening phase, which is largely compensated for by grain cross-sectioning in the considered zone. The lowest measured value of microhardness for the HT-1 sample is 98 HV0.1 and is located symmetrically at a distance of 3.5 mm from the center of the weld. Most probably, this area is characterized by the lowest ratio of thermal evolution of the strengthening phase to the occurring grain refinement. An increase in microhardness can be observed from this area as it moves away from the center of the weld. This is the result of the decreasing temperature influence on the welded material. The application of thermal treatment after the joining process (HT-2) causes the elimination of differences in the hardness values on the cross-section of the joint.

The heat-affected zone (HAZ) is not possible to observe in the HT-2 sample due to grain recrystallization during post-weld heat treatment. The boundary between the TMAZ and HAZ zones is difficult to define, although the method of detection of the external TMAZ boundary based on the angular distortion of grains has been developed by Woo et al. [48]. Determination of the boundary between the base material and the heat affected zone is also difficult because the variability of the microstructure between these areas is insignificant. Measurements of the external HAZ border require the use of thermocouples to determine temperature limits, complicating the investigation. The HAZ microstructure in the HT-0 and HT-1 joints is characterized by large, elongated grains oriented in the direction of the plate’s rolling. The elongation of grains in TMAZ results from plastic deformation, but to a lesser extent than in the NZ. On the other hand, there is no plastic deformation in HAZ except the evolving heat shapes the microstructure in this region, causing the grain size to increase in relation to TMAZ. The average grain size in HAZ is more similar to the grain size of the base material than to the grain size in the TMAZ. In the case of joint in the HT-2 state, it is not possible to separate the HAZ area from the BM, due to the homogeneity of the grain size.

**Figure 8:**
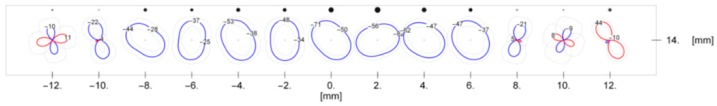
The contours of surface stresses determined along the long axis of the HT-0 sample.

**Figure 9:**
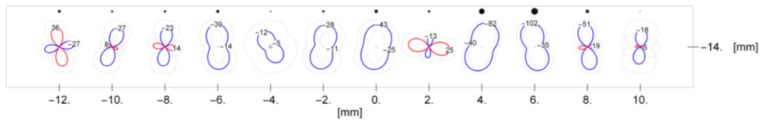
The contours of surface stresses determined along the long axis of the HT-1 sample.

**Figure 10:**
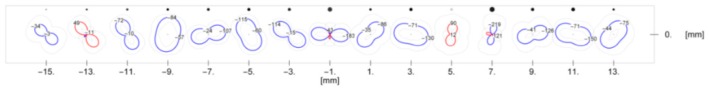
The contours of surface stresses determined along the long axis of the HT-2 sample.

To measure residual stresses, each sample was placed in the diffractometer so that the center of the joint was as close as possible to the 0 mm horizontal coordinate. For the prepared contours of residual stresses, above the measurement points, additional black discs were used, the size and clarity of which represent the relative size and relative uncertainty of the calculated principal stress components (Figure 2). The values of the principle stress components for each sample together with contours of surface stresses distribution are shown in Table 4, Table 5 and Table 6 and in Figure 8, Figure 9 and Figure 10 respectively. The values of mean stresses for each sample are presented in Table 7.

In the region of the weld, the material exhibits compressive stresses, close to the beneficial isotropic state σ_1_ = σ_2_. At a distance 8 mm from the weld, small tensile stresses appear, and the nature of the stress tensor becomes more anisotropic.

For this sample, the variation in residual stresses shows a low correlation with the location of the weld. The HT-1 sample is characterized by the lowest average values of stresses from among three examined joints.

Similarly to the HT-1 sample, the recorded variation in residual stresses shows a smaller correlation with the location of the weld than in the case of the HT-0 sample. The determined values of stresses of HT-2 sample are the highest observed for the investigated joint with σ_1_ = −53 MPa and σ_2_ = −59 MPa. In the absence of heat treatment, the FSW process affects the microstructure of the welded material, determining its hardness and state of stresses, characteristics of which remain significantly correlated with the course of the weld. An increase in the hardness and formation of compressive residual stresses in the nugget zone of the HT-0 sample is visible. However, heat treatment of a material is a process that dominates the properties of samples from the AA2519 alloy, leading to homogenization of its microstructure and increase of hardness to a higher level than the one obtained in the stir zone of samples HT-0 and HT-1. Simultaneously, the state of stresses introduced by heat treatment of the material (anisotropic compressive stresses) is least favorable than that obtained only by FSW welding (close to isotropic compressive stresses in the weld area). The conducted research indicates that the best material properties: homogenous microstructure and uniform distribution of microhardness and compressive state of residual stresses, were obtained for the HT-2 series samples subjected to heat treatment after the FSW joining process.

## 4. Conclusions

Based on the analysis of microstructure and analysis of FSWed AA2519 (under three different conditions) following conclusions can be drawn:

1. The post-weld heat treatment of AA2519 FSW joint in the form of precipitation hardening provides a number of favorable properties, such as the uniform distribution of microhardness values and residual stresses in the joint. However, grain overgrowth in the stir zone has been reported due to heat treatment.
2. The investigated alloy, in non-heat treated condition (HT-0) undergoes the most severe changes in terms of microhardness during the FSW process. Due to grain refinement in the stir zone, the microhardness increases from 92 HV0.1 (base material value) to 108 HV0.1 on the advancing side and 100 HV0.1 on the retreating side. At the same time on the retreating side of the heat-affected zone, the lowest value of microhardness is reported—72 HV0.1.
3. During FSW, the alloy in heat-treated condition (HT-1) suffers slight reduction in microhardness values due to overaging of the strengthening phase, but values for the stir zone are higher than in the case of sample HT-0 and are equal to about 105-110 HV0.1. Additionally, in terms of residual stress, the sample HT-1 is characterized by the lowest average values of stresses from among the three examined joints.

## Figures and Tables

**Table 1:** Chemical composition of AA2519 to be weld.

Si	Fe	Cu	Mg	Zn	Ti	V	Zr	Sc	Al
0.06	0.08	5.77	0.18	0.01	0.04	0.12	0.2	0.36	Base

**Table 2:** The dimensions of the used MX Triflute tool.

Shoulder Profile	Spiral
Shoulder diameter	19 mm
Pin profile	Threaded and tapered with three spiral flutes
Pin length	4.8 mm
Pin diameter	6.5–8.7 mm

**Table 3:** The designation of the samples.

Designation	State of The Sample
HT-0	AA2519 welded in non-heat treated condition
HT-1	AA2519 welded in heat-treated condition
HT-2	AA2519 welded in non-heat treated condition subjected to the post-welded heat treatment

**Table 4:** The values of the principle σ_1_ and σ_2_ stress components and the directions (γ angle) of the σ_1_ action on the sample’s surface, determined for the HT-0 sample.

	X [mm]	y [mm]	γ [deg]	u (γ) [deg]	σ_1_ [MPa]	u (σ_1_) [MPa]	σ_2_ [MPa]	u (σ_2_) [MPa]
1	−12.0	14.0	79.95	12.99	−9.94	6.08	11.00	5.76
2	−10.0	14.0	71.66	12.11	−22.42	5.43	3.76	6.24
3	−8.0	14.0	33.02	17.69	−43.87	6.57	−27.55	8.76
4	−6.0	14.0	97.16	26.53	−37.15	7.59	−24.67	5.73
5	−4.0	14.0	62.70	23.81	−52.77	6.91	−37.85	7.31
6	−2.0	14.0	88.25	25.46	−48.36	7.94	−33.73	7.44
7	0	14.0	45.57	16.19	−70.80	6.20	−49.65	8.13
8	2.0	14.0	72.81	12.26	−55.71	10.10	−81.64	7.59
9	4.0	14.0	27.74	20.42	−62.48	7.37	−46.54	9.69
10	6.0	14.0	51.69	34.73	−46.63	6.74	−37.47	7.11
11	8.0	14.0	15.79	11.91	5.42	7.04	−20.73	8.59
12	10.0	14.0	25.23	18.89	8.20	6.99	−9.43	7.8
13	12.0	14.0	57.67	5.86	43.76	6.84	−10.26	6.58
Mean			55.65 ± 3.69		−28.62 ± 1.90		−24.12 ± 1.99	

**Table 5:** The values of the principle σ_1_ and σ_2_ stress components and the directions (γ angle) of the σ_1_ action on the sample’s surface, determined for the HT-1 sample.

	x [mm]	y [mm]	γ [deg]	u (γ) [deg]	σ_1_ [MPa]	u (σ_1_) [MPa]	σ_2_ [MPa]	u (σ_2_) [MPa]
1	−12.0	14.0	78.70	7.12	35.63	13.92	−27.28	9.36
2	−10.0	14.0	22.36	11.10	8.19	8.26	−27.02	9.35
3	−8.0	14.0	97.17	11.86	−23.22	9.62	14.19	7.44
4	−6.0	14.0	85.62	14.07	−38.78	7.58	−13.66	7.73
5	−4.0	14.0	57.29	43.05	−12.41	6.99	-4.88	6.62
6	−2.0	14.0	101.79	21.99	−27.81	5.68	−11.06	6.62
7	0	14.0	102.70	20.37	−42.86	7.75	−24.56	8.10
8	2.0	14.0	98.81	8.50	−13.47	6.10	25.36	7.74
9	4.0	14.0	19.88	7.63	−40.09	6.52	−81.51	7.56
10	6.0	14.0	77.35	7.23	−101.90	12.51	−35.50	10.17
11	8.0	14.0	82.10	6.09	−51.39	9.32	19.37	13.36
12	10.0	14.0	96.76	19.41	−17.56	10.20	5.21	13.69
Mean			72.16 ± 2.83		−26.63 ± 2.27		−14.64 ± 2.40	

**Table 6:** The values of the principle σ_1_ and σ_2_ stress components and the directions (γ angle) of the σ_1_ action on the sample’s surface, determined for the HT-2 sample.

	X [mm]	y [mm]	γ [deg]	u (γ) [deg]	σ_1_ [MPa]	u (σ_1_) [MPa]	σ_2_ [MPa]	u (σ_2_) [MPa]
1	−15.0	0.0	38.73	25.04	−34.28	18.86	−2.90	14.64
2	−13.0	0.0	48.79	16.40	49.19	17.18	−10.83	18.39
3	−11.0	0.0	59.33	13.06	−72.40	18.65	−9.62	17.52
4	−9.0	0.0	103.24	32.48	−83.76	14.93	−56.67	14.41
5	−7.0	0.0	78.19	11.68	−23.95	21.63	−106.60	20.48
6	−5.0	0.0	62.70	15.17	−115.20	24.95	−60.45	18.49
7	−3.0	0.0	34.18	8.32	−113.60	16.65	−14.59	18.38
8	−1.0	0.0	94.04	15.23	43.12	75.40	−183.40	20.90
9	1.0	0.0	47.98	29.99	−35.21	36.08	−85.56	29.55
10	3.0	0.0	101.90	20.84	−71.23	30.30	−129.70	27.53
11	5.0	0.0	99.21	41.96	90.07	72.00	12.10	80.33
12	7.0	0.0	103.45	17.87	−218.60	216.40	121.40	89.81
13	9.0	0.0	75.27	31.20	−40.85	28.56	−125.60	42.67
14	11.0	0.0	103.86	17.18	−71.29	34.04	−150.30	24.55
15	13.0	0.0	42.21	40.00	−43.70	30.07	−75.04	25.26
Mean			65.03 ± 4.28		−53.2 ± 6.16		−58.95 ± 5.52	

**Table 7:** The mean stress value for investigated joints.

Sample	Mean Stress [MPa]	
HT-0	*σ*_1_ = −28.62 ± 1.90	*σ*_2_ = −24.12 ± 1.99
HT-1	*σ*_1_ = −26.63 ± 2.27	*σ*_2_ = −14.64 ± 2.40
HT-2	*σ*_1_ = −53.26 ± 6.16	*σ*_2_ = −58.95 ± 5.52
